# Co-Encapsulation of Epigallocatechin-3-Gallate and Vitamin B12 in Zein Microstructures by Electrospinning/Electrospraying Technique

**DOI:** 10.3390/molecules28062544

**Published:** 2023-03-10

**Authors:** Ana F. Couto, Maéna Favretto, Raphael Paquis, Berta N. Estevinho

**Affiliations:** 1 LEPABE-Laboratory for Process Engineering, Environment, Biotechnology and Energy, Department of Chemical Engineer, Faculty of Engineer, University of Porto, Rua Dr. Roberto Frias, 4200-465 Porto, Portugal; 2 ALiCE—Associate Laboratory in Chemical Engineer, Faculty of Engineering, University of Porto, Rua Dr. Roberto Frias, 4200-465 Porto, Portugal; 3 ENSCM—Ecole Nationale Supérieure de Chimie de Montpellier, 8 Rue de l’Ecole Normale, CEDEX 5, 34296 Montpellier, France

**Keywords:** EGCG, electrospinning, microencapsulation, vitamin B12, zein

## Abstract

EGCG is a catechin known for its antioxidant and anti-inflammatory characteristics. Vitamin B12 is an essential vitamin found in animal-derived products, and its deficiency may cause serious health problems such as anemia. The effectiveness of both catechin and vitamin B12 depends on their stability and bioavailability, which can be lost during industrial processes due to degradation when exposed to external factors. A potential solution to this issue is the microencapsulation, which protects the compounds from external agents. The current study aims to microencapsulate EGCG and vitamin B12 in a polymer matrix of biological origin, zein. Microencapsulation was performed using an electrospinning technique, and different concentrations of zein (1–30% *w*/*v*) and active compound (0.5–5% *w*/*w*) were tested, resulting in the production of micro/nanoparticles, fibers, or the mixture of both. The microstructures were analyzed and characterized in terms of morphology, release profile and kinetics, and encapsulation efficiency. High encapsulation efficiencies were obtained, and the highest were found in the samples with 1% *w*/*w* of active substance and 30% *w*/*v* of zein. Controlled release studies were conducted in deionized water and in an ethanolic solution, and five kinetic models were applied to the release profiles. The results indicated that the Weibull model was the best fit for the majority of results.

## 1. Introduction

Nowadays, there is a growing concern about health and the type of products consumed by the society. The demand for food products, supplements, and cosmetics containing natural bioactive ingredients such as antioxidants, namely catechins, and vitamins, such as vitamin B12, is increasing [1,2,3].

Catechins are natural polyphenolic compounds (polyphenols) and are a highly diversified group synthesized by plants [4,5,6]. Epigallocatechin-3-gallate (EGCG) is the most abundant and bioactive catechin, making up to 59% of total catechins in green tea [7,8]. In recent years, studies have confirmed that EGCG has health benefits, including as an antioxidant, antitumor, anti-inflammatory, antibacterial, antiviral, and protection against UV radiation [9,10,11,12]. ECGC has also been shown to promote mitochondrial biogenesis and improve cellular function [13]. Due to EGCG’s appealing characteristics and health benefits, the interest in its use as a dietary supplement and food preservative is increasing, and it can be found in a variety of foods such as ready-to-eat meals, cereals, bakery goods, salad dressings, margarine, oils, and beverages [9]. However, these interesting qualities depend on the bioavailability and stability of the EGCG in the human body. In general, catechins present low bioavailability after oral administration because they are highly unstable to changes in pH and temperature and are easily transformed into non-epi structures [11,14].

Vitamins are a heterogenous and diverse group of compounds that are required for appropriate physiological function (micronutrient), but most are not generated by the body, at least not enough to meet human daily needs [15,16,17,18]. Vitamin B12 is a water-soluble vitamin that belongs to the B-complex and is also known as Cobalamin (Cbl). It has a complex structure and is the only vitamin containing a metal, central cobalt atom [19,20,21]. Vitamin B12 is synthesized by certain bacteria in the gastrointestinal tract of animals and is subsequently absorbed by the host animal. Therefore, it can only be found in animal-derived foods, with the liver being the most abundant source [22]. There are no bioactive forms of vitamin B12 from plant sources [23]. Vitamin B12 plays an essential role in various body functions, such as normal growth and development (especially in children), normal production of red blood cells (in conjunction with folate), nerve system function, DNA synthesis, processing and burning of fat and carbohydrate, and participates in the conversion of homocysteine to methionine [18,20,21]. Lack or malabsorption of this vitamin can cause health problems, such as anemia [2]. Regarding stability, aqueous solutions and crystalline forms of Cbl are considered stable when protected from light exposure [24]. However, a portion of vitamin B12 is degraded and loses activity during cooking (at temperatures higher than 120 °C) and storage [2]. It is also susceptible to degradation in the presence of compounds such as ascorbic acid, thiamine, niacin, strong acids, metals, and UV light [25].

Given the aforementioned information, it is critical to ensure the stability, bioactivity, and bioavailability of EGCG and Vitamin B12 when added to food products or supplements, as well as to manage their release to ensure they reach the intended target. Furthermore, since EGCG has an astringent and bitter taste, it is necessary to mask its flavor, as it can be unpleasant when consumed [14,26]. One way to address these issues is through microencapsulation, which presents several advantages, such as enhancing the stability of the bioactive ingredients, protecting them from external factors, facilitating their handling and storage, and preserving organoleptic qualities, among others [19,27].

Microencapsulation is an innovative and promising technology that involves the encapsulation of small particles (such as gases, liquids, or solids) in a polymeric matrix, resulting in microstructures with a size distribution ranging from 1–5000 μm and different morphologies and internal structure [1,28,29,30]. This technique is widely used across various industries, including food, nutraceutical, pharmaceutical, agricultural, cosmetics, and textile industries [1,31]. Many antioxidants, such as caffeine, green tea polyphenols, β-carotene, and vitamins like folic acid have been reported to be encapsulated using this technology [32,33].

Electrohydrodynamic processes (EHD) are a promising option for microencapsulation due to their ability to overcome disadvantages associated with traditional encapsulation techniques, such as the need for high temperatures and extreme pH conditions. EHD processes use high-voltage electrostatic fields to charge the surface of a polymer solution jet, creating microstructures with a high surface area to volume ratio and high porosity [34,35,36]. EDH processes can operate in two similar methods: electrospinning, which produces microfibers, and electrospraying, which similarly creates micro/nanoparticles [35]. The main difference between these two methods lies in the polymer concentration, with electrospraying occurring when the concentration is low enough to destabilize the jet and form fine-charged droplets [34,36]. However, given the complexity of these processes, there are several conditions and parameters that can be adjusted to obtain microstructures with different characteristics.

As stated in Huang et al., 2021 [30], electrospinning has been used to encapsulate various bioactive compounds, including hydrophobic compounds such as lipids, vitamins, curcumin, and essential oils, as well as hydrophilic compounds such as anthocyanin and gallic acid, enzymes, and probiotic. Recent studies revealed that electrospun materials can be used for various applications such as drug delivery, tissue engineering, wound healing, and biosensors. For instance, electrospun fibers can be employed to produce scaffolds that mimic the natural tissue structure and can also be used to create dental materials for tissue regeneration and repair [13,37,38,39].

However, to the best of our knowledge, there is a lack of studies that have co-encapsulated vitamins and polyphenols using electrodynamic techniques.

In this study, EGCG and vitamin B12 were encapsulated in zein microstructures produced by electrospinning, with zein as a wall material in order to characterize and evaluate the morphology of the produced structures, the release behavior (mathematical models were fit to the data) and encapsulation efficiency.

The choice of zein as the encapsulating agent was based on previous studies and its advantages and disadvantages as a biopolymer [2,40]. Zein is a prolamin protein extracted from the corn endosperm, making up 35–60% of total proteins in corn. Its hydrophobic properties, low moister absorption, high thermal resistance, and oxygen barrier properties make it a popular choice for processing and encapsulating compounds [35,40,41]. However, due its high content of hydrophobic amino acids, zein is insoluble in water and soluble in specific solvents, such as aqueous alcohols, alkaline solutions, and aqueous solution with high concentrations of urea or anionic detergents [35,42]. Zein has been widely used in various industries, including pharmaceuticals and food, as it has been deemed a safe food ingredient by the Food and Drug Administration (FDA) [40]. For instance, Moomand and Lim, 2015 [43], proposed that zein could be a promising matrix for encapsulating omega-3-rich-fish-oil through electrospinning/electrospray. Additionally, Gómez–Mascaraque et al., 2017 [44], reported on the potential of electrospray zein microstructures to enhance the bioaccessibility of carotenoids after digestion.

## 2. Results and Discussion

The main goal of the study was to produce biopolymeric microstructures with high encapsulation efficiencies, using electrospinning/electrospraying techniques and incorporating a catechin, EGCG, and a vitamin, vitamin B12. The effect of zein, EGCG, and vitamin B12 concentrations in the microstructures morphology and release behaviour were evaluated.

### 2.1. Characterization of the Zein Microstructures: Scanning Electron Microscopy

**Figure 1:**
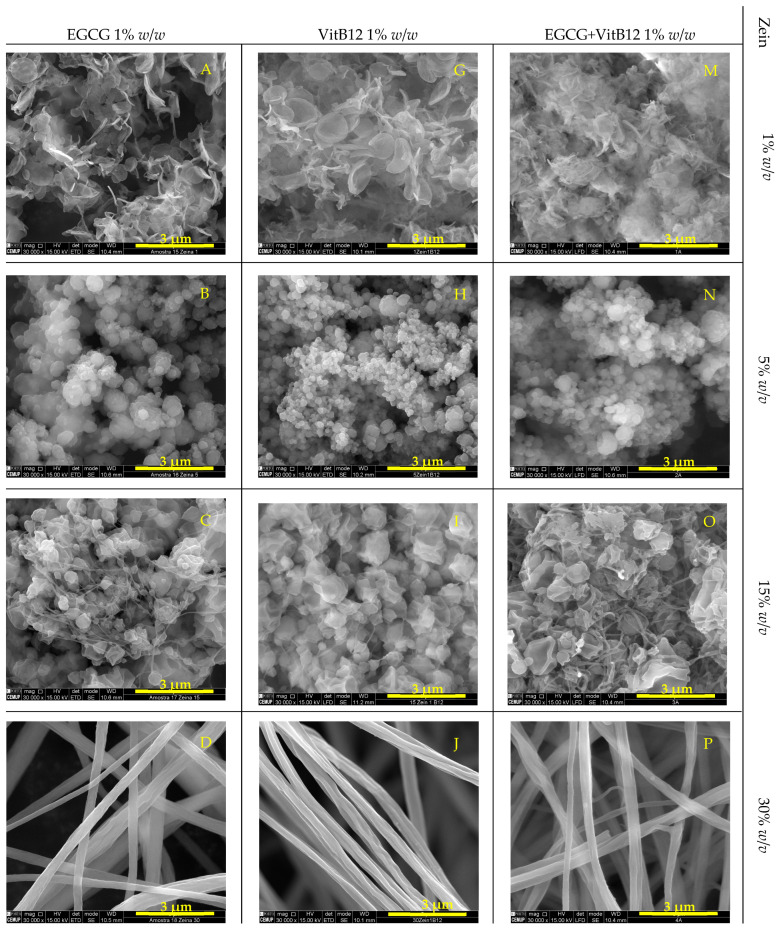

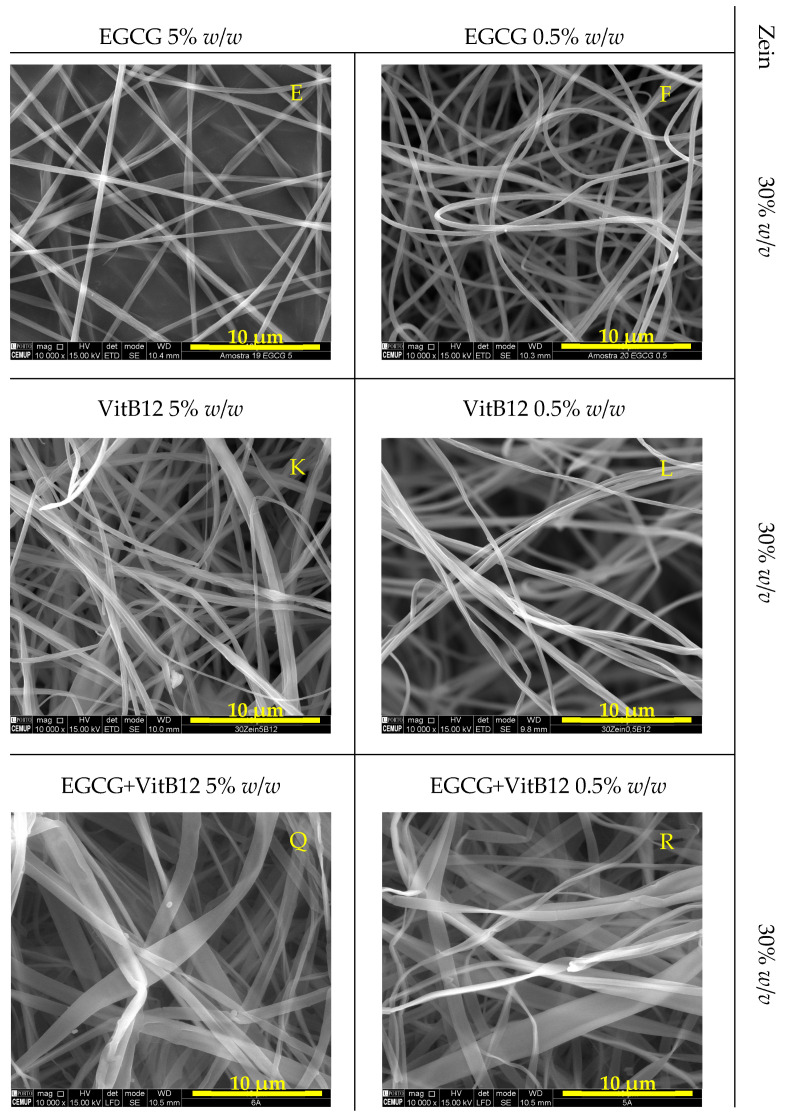
SEM images of microstructures (pump controlled samples) containing different concentrations of zein, EGCG, and VitB12. Magnification of 30,000× for (**A**–**D**,**G**–**J**,**M**–**P**) and 10,000× for (**E**,**F**,**K**,**L**,**Q**,**R**), beam intensity (HV) 15.00 kV, distance between the sample and the lens (WD) less than 10.6 mm, scale bar of 3 μm for (**A**–**D**,**G**–**J**,**M**–**P**) and 10 μm for (**E**,**F**,**K**,**L**,**Q**,**R**).

**Figure 2:**
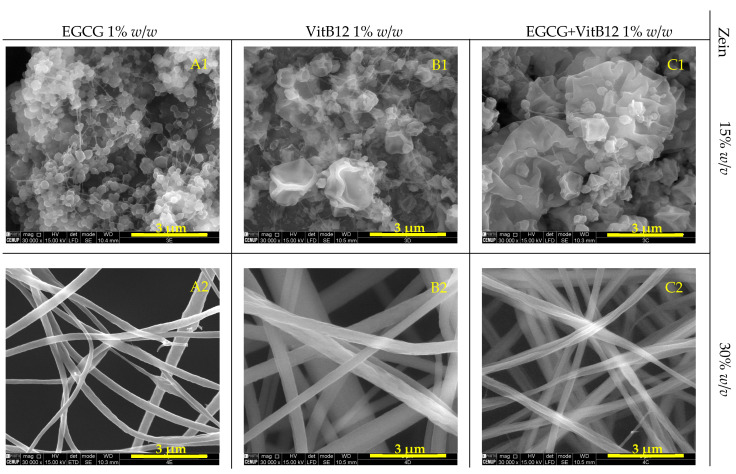
SEM images of microstructures (pressure controlled samples) containing 15% *w/v* (**A1**–**C1**) and 30% *w*/*v* zein (**A2**–**C2**), and EGCG, VitB12 and (EGCG + VitB12). Magnification of 30,000×, beam intensity (HV) 15.00 kV, distance between the sample and the lens (WD) less than 10.6 mm, and scale bar of 3 μm.

Zein microstructures containing EGCG, VitB12, and the mixture of both were evaluated using scanning electron microscopy (SEM) (Figure 1 and Figure 2).

Figure 1 and Figure 2 show that as the zein concentration increases, the morphology of the structures tend to change. The highest concentration of zein used (30% *w*/*v*) formed fibers, while the intermediate concentration (15% *w*/*v*) produced a mixture of fibers and particles. The lowest concentration of zein (1 and 5% *w*/*v*) resulted in different types of micro/nanoparticles.

In a study conducted by Coelho et al., 2021 [2], the production of zein microstructures containing vitamin B12 by electrospinning was investigated. In the study, the parameters used were similar to those used in the present work. Higher zein concentration promoted the formation of fibers, while lower concentrations of zein resulted in the formation of microparticles. The results of this study were similar to those obtained in the present work.

Samples containing 1% *w*/*v* zein display a unique shape characterized by small and flattened particles that are thin and porous, creating structures similar to small films. A study conducted by Coelho et al., 2022 [40], reported the formation of heterogenous matrix microstructures in samples containing 1% *w*/*v* zein, using electrospinning with a flow rate of 0.3 mL/h, a voltage of 20 kV and 7 cm of tip-to-collector distance to encapsulate vitamin B9. However, this was not observed in the samples obtained in this study. Samples produced with 5% *w*/*v* zein, on the other hand, show small, round particles with rough surfaces. These microstructures were produced using a low polymer concentration leading to the process functioning as electrospray instead of electrospinning, resulting in fine droplets instead of fibers [2]. As the concentration of zein increases, fibers begin to form instead of microparticles, and a mixture of microparticles become evident. The microparticles in samples containing 15% *w*/*v* zein have an irregular surface, as if they possess an outer layer that has imploded as the solvent evaporated [33]. Finally, the samples with 30% *w*/*v* zein exhibit the formation of homogeneous, continuous fibers with a slightly rough surface. The shape of these fibers ranges from tubular (samples containing 30% *w*/*v* zein + EGCG or VitB12) to ribbon-like (samples containing 30% *w*/*v* zein + (EGCG + VitB12)).

The transition from the formation of microparticles to fibers is likely due to the greater resistance of the high-concentration solutions to be drawn towards the collector or due to the electrical conductivity of the solution [45] The morphology of the samples was not altered by the type or concentration of the core material, indicating that the type and concentration of encapsulating agents are the determining factors in forming different structures through electrospinning. However, the co-encapsulating microfibers seem to have a greater width compared to fibers produced with only one component.

Samples obtained through pressure (Figure 2) exhibited similar structures and shapes under the same zein and active core concentrations and procedure conditions.

The diameter of the particles and the width of the fibers were measured using the SEM images. Overall, particles formed with 1%, 5%, and 15% *w*/*v* zein have a diameter ranging from 0.2 to 0.8 µm. No differences in size were observed when compared between the type of the core. The fibers formed with 30% *w*/*v* zein have a width between 0.4 and 1 µm. The largest width is observed in fibers with the mix core, at every core concentration (0.5%, 1% and 5% *w*/*w* EGCG + VitB12).

### 2.2. In Vitro Release Assays

**Figure 3:**
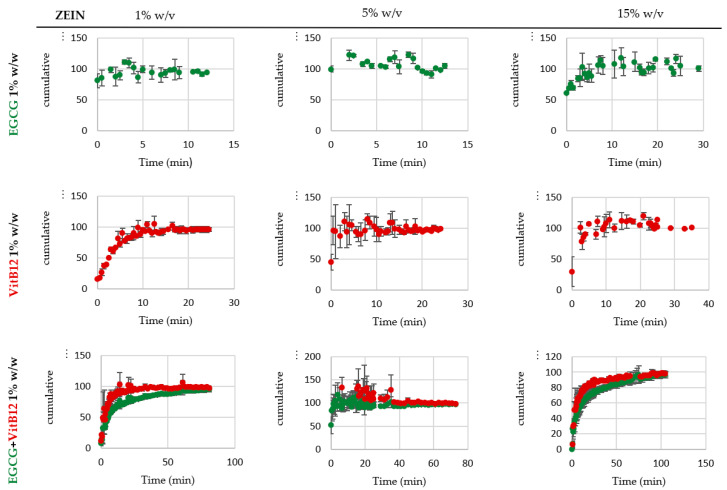
In vitro release profiles, % of EGCG/VitB12/(EGCG+VitB12) normalized by the total amount released, in H_2_O of the microstructures (electrospinning) loaded with 1% *w*/*w* of active compounds and 1%, 5% and 15% *w*/*v* zein.

**Figure 4:**
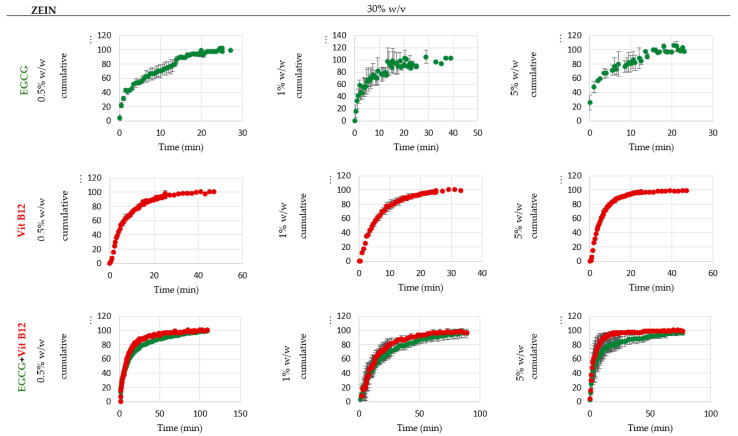
In vitro release profiles, % of EGCG/VitB12/(EGCG + VitB12) normalized by the total amount released, in H_2_O of the microstructures (electrospinning) loaded with 0.5%, 1%, and 5% *w*/*w* of active compounds and 30% *w*/*v* zein. In a first approach, it is evident that most samples present a similar release behavior, with the exception of EGCG samples containing 1% and 5% *w*/*v* zein, which show immediate release. For all other samples, it is possible to distinguish two different zones: the release zone, which is identified by the increase of the core release, and the stabilization zone, characterized by a stabilization of the release.

The release behavior is largely affected by the interactions between the polymeric matrix, the solvent, and the core material, as well as other factors such as the type of microstructure (particles, fibers, or films). There are several release mechanisms that vary based on the encapsulating agent, preparation method, and the environment in which the release occurs. It can be based on a single mechanism or a combination of mechanisms, including processes of diffusion, degradation, and biodegradation, dilatation (gel formation and swelling), melting, and osmosis [46]. Figure 3 and Figure 4 show the release profiles obtained in deionized water from the pump-controlled samples containing different concentrations of zein and core material, with the release percentage (amount release at a time t normalized by total release amount) plotted over time. The release profiles in 50% ethanol are not displayed since zein is soluble in aqueous ethanol solutions, resulting in the near-immediate release of the core.

Upon analyzing the graphs, it can be seen that as the zein concentration increases, the release time also tends to increase, regardless of the type of core material. This means that microparticles have a quicker release compared to fibers in deionized water. This result may suggest that as the zein concentration increases and the type of microstructure changes, the initial amount of core released decreases and becomes slower and prolonged. Coelho et al., 2021 [2], reported similar findings with zein microstructures loaded with vitamin B12 and released in ethanol (concentrations of zein ranging from 3 to 30% *w*/*w*, dissolved in ethanol 60% *v*/*v*, concentrations of vitamin B12 ranging from 1 to 10% *w*/*w*, the flow rate used was 0.2 mL/h or 0.3 mL/h, and a 7 cm of distance from the tip to the collector).

A comparison of release profiles for samples with different EGCG concentration reveals that as EGCG concentrations decrease, the time required to release all the content from the microstructures increases. Therefore, samples with 0.5% *w*/*w* EGCG will have a longer release time than those with 1% and 5% *w*/*w*. The same phenomenon also happens for samples loaded with VitB12 and the mixture of catechin and vitamin B12. This result is in line with Hosseini et al., 2021 [45], who reported a similar behavior with zein fibers loaded with rosemary essential oil, resulting in a large initial burst. When comparing the single samples with the co-encapsulated samples, it is interesting to note that the release time is longer when catechin is mixed with vitamin B12 than when they are isolated.

The pressure-controlled samples showed similar release behaviors and release profiles, considering this the profiles are not presented.

To analyze the release data, five kinetic models were adjusted (as shown in Table 1 and Table 2). A review of the parameters reveals that the Weibull model and the Baker–Lonsdale model showed the best fit to the experimental results, with correlation coefficients ranging from 0.761 to 0.994 and 0.845 to 0.998, respectively. Among these two models, the Weibull model was found to adjusts to a greater number of results as the Baker–Lonsdale model only adjusts to measurements up to 80% of the total release. This conclusion applies to the majority of the samples, with an exception for Z:30:0.5, where the Korsmeyer–Peppas and Baker–Lonsdale had a better fit, and for Z:30:B:5, where the Zero order and Baker–Lonsdale models provided the best fit.

The Baker–Lonsdale model is derived from the Higuchi model and is commonly used for the linearization of the release data from microcapsules and spherical matrices. This model is associated with releases based on a diffusion mechanism [47]. However, it was observed that the model fit well to releases from a variety of microstructures, including spherical microparticles and fibers. The constant obtained from this model, *K*, was found to have lower values for slower releases, such as those in the samples with the mixture of active agents (EGCG + VitB12). From the Korsmeyer–Peppas model, it is possible to retrieve the values of the parameter *n* (release exponent) and the constant *K_k_*. The parameter *n* defines the mechanism responsible for the release of the core [29]. The samples that followed a “Fickian Diffusion” (*n* < 0.43 for the microparticles; *n* < 0.45 for the fibers) were Z:1:B:1, Z:1:EB:1, Z:15:E:1, Z:15:B:1, Z:30:E:0.5, Z:30:E:5, and Z:30:EB:0.5. The remaining samples followed a “Super Case-II transport” (*n* > 0.85 for the microparticles; *n* > 0.89 for the fibers), except for the Z:15:EB:1 and Z:30:EB:1 that followed an “Anomalous Transport” (0.43 < *n* < for the microparticles; 0.45 < *n* < 0.89 for the fibers). In the super case-II transport, the sorption is entirely controlled by stress-induced relaxations and the anomalous transport occurs involving both Fickian diffusion and polymer chain relaxation.

With these results, it is possible to conclude that the mechanism associated with the core release from the microparticles is essentially the “Fickian Diffusion” and the fibers are “Super Case-II transport.”

Lastly, the Weibull model proved to be the most adequate for analyzing the matrix-type (active compound distributed through the encapsulating agent) profile release, which is the case for the structures obtained in the present work. The parameter *β*, which is related with the shape of the release curve, was less than 1 for all samples except Z:30:B:1, where the *β* value was 1.051. Thus, when *β* < 1, the curves showed an exponential-like shape but with a steeper increase when compared to the ones with *β* = 1. When *β* > 1, the profile displayed a sigmoidal shape.

Coelho et al., 2021 [2], conducted a study on encapsulating vitamin B12 in zein using electrospinning and spray drying techniques and obtained similar results for nearly all the samples.

### 2.3. Entrapment Efficiency

One of the most important quality parameters in microencapsulation of active compounds is the encapsulation/entrapment efficiency (EE). EE indicates the amount of substance (bioactive compound) that is successfully entrapped within the particles or fibers [48]. The encapsulation efficiencies of EGCG, VitB12, and EGCG + Vit12 in electrospun zein microstructures from blend solutions of the encapsulating agent and active ingredient are described in Table 3. There are different methods and approaches to determine it [49]. In the present study, and considering the specifications of the experiment, the encapsulation efficiency can be calculated considering the release profiles as described in Materials and Methods.

The encapsulating efficiencies range from 0.309% to 100% (the EE with * are above 100% due to analytical errors and a possible small interference of the encapsulating agent in the absorbance measurement). Most of the samples present an EE above 70%, which is considered a high efficiency. Similar results were obtained for the pressure-controlled samples.

The highest EE is observed in the samples containing 30% *w*/*v* zein and 1% *w*/*w* of core material. These results suggest that zein fibers were the best microstructure for encapsulating either catechin and vitamin alone or co-encapsulated at all concentrations tested, with the optimal concentration being 1% *w*/*w*. When comparing the EE of the samples with a simple core and the samples with co-encapsulation, the results are similar between samples. Analogous results were reported by Bucurescu et al., 2018 [49], while developing a Gum Arabic matrix by spray drying to microencapsulate curcumin; the highest encapsulation efficiencies were achieved in the particles prepared with higher amounts of encapsulating agents.

The microstructures prepared with zein by an electrodynamic process have shown a high potential for encapsulating and protecting photosensitive bioactive compounds such as the polyphenols (EGCG) and vitamins (vitamin B12). So, this work shows that it is possible to co-encapsulate polyphenols and vitamins through an electrospinning/electrospraying process. Therefore, depending on the pretended application (food supplementation, nutraceutical, cosmetic or pharmaceutical), one determined formulation (microparticles/fibers) should be considered.

## 3. Materials and Methods

### 3.1. Materials

The zein (grade Z3625) powder was purchased from Sigma–Aldrich (St. Louis, MI, USA) and used as received without further purification. EGCG from green tea and vitamin B12 were both purchased from Sigma–Aldrich. The 70% *v*/*v* aqueous ethanol solution was purchased from VWR BDH-chemicals and used as the solvent. Deionized water—Milli Q water, resistivity of 18.2 MΩ/cm at room temperature (~23 °C)—was used for the in vitro release tests and the construction of calibration curves.

### 3.2. Microstrutures Production

Two solutions were prepared: the zein solutions and the active compound solutions (EGCG and vitamin B12), both in 70% *v*/*v* ethanol. Zein solutions were prepared with different concentrations ranging from 1% to 30% (*w*/*v*, weight of zein/volume of solvent)—Table 1. The concentrations of EGCG and vitamin B12 used range between 0.5% and 5% (*w*/*w*, weight of EGCG or VitB12/weight zein), as shown in Table 4. Samples containing a mixture of EGCG and VitB12 (co-encapsulation) were also prepared, and their concentrations are also listed in Table 4. The EGCG, vitaminB12, and mixture solutions were added to the polymer solutions prior to the electrospinning process, as indicated in Table 1.

Different microstructures were obtained using an electrospinning experimental set-up equipped with a variable high-voltage (0–20 kV) power supply and was supplied by Spraybase^®^ (Dublin, Ireland). The solutions were pumped under a steady-state flow rate using an electric pump and a stainless-steel needle (22 G) positioned 5 cm away from a metal collector plate. A voltage of 20 kV was applied during the process.

In addition, some zein samples were also produced using a pressure-based method with varying pressure values ranging from 0.0025 to 0.064 bar. All other conditions remain the same as the pump-controlled samples. The optimal conditions were considered and the process occurred at room temperature (approximately 23 °C). The resulting microstructures were scraped from the collector and placed in plastic containers.

### 3.3. Microstrutures Morphology

The morphology and shape of the microstructures were evaluated in several fragments of the samples by scanning electron microscopy (SEM)—Fei Quanta 400 FEG ESEM/EDAX Pegasus X4M equipment (Eindhoven, The Netherlands) at Centro de Materiais da Universidade do Porto (CEMUP), Porto, Portugal.

### 3.4. In Vitro Release Assays

The in vitro release assays were performed for all samples in deionized water and 50% *v*/*v* ethanol to simulate commonly used industrial formulations (aqueous and ethanolic solutions) in food, cosmetic, and pharmaceutical products. The assays involved placing a sample in a cuvette with water or ethanol with magnetic stirring, and the absorbance at a wavelength of 274 ± 2 nm (EGCG) and 361 ± 2 nm (VitB12) was measured using a NanoDrop One-C spectrophotometer (Thermo Fisher Scientific, Waltham, MA, USA). The release of the core was determined by continuously measuring the absorbance until the value of absorbance stabilized. All the assays were made in triplicate.

The concentration of EGCG and VitB12 were determined using linear calibration curves in both deionized water and 50% *v*/*v* ethanol. An EGCG calibration curve was constructed using eight standard solutions ranging from 0.0001 mg/mL to 0.1 mg/mL, and a vitamin B12 calibration curve was made with 10 standard solutions ranging from 0.002 mg/mL to 0.05 mg/mL. Both calibration curves had correlation coefficients higher than 0.998. All the measurements were made in triplicate.

To the release profiles, those obtained for each sample (pump-controlled samples) were adjusted five mathematical models, as described in Estevinho and Rocha, 2017 [46]. These models, including Zero Order, First Order, Korsmeyer–Peppas, Weibull, and Baker–Lonsdale are commonly used to characterize the release of active ingredients and to optimize process parameters for the design of structures with specific properties [29].

The kinetics of zero order (constant release rate) happens when the core is a pure substance (Equation (1)), and the first order occurs when the inside core is a solution (Equation (2)) [50].

(1) $$Q_{t}=Q_{0}+K_{0}t$$

(2) $$Q_{t}=Q_{0}e^{-K_{1}t}$$

where *Q_t_* is the cumulative amount of the active ingredient released at time *t*, *Q*_0_ is the initial amount in the solution, and *K*_0_ and *K*_1_ are the zero-order and first-order release constants, respectively.

Although these simple models provide some characterization of the release of active substances, more accurate descriptions of the release phenomenon require the use of more complex models, such as the Korsmeyer–Peppas (Equation (3)), Weibull (Equation (4)), and Baker–Lonsdale models (Equation (5)).

(3) $$\frac{Q_{t}}{Q_{\infty }}=K_{k}t^{n}$$

(4) $$\frac{Q_{t}}{Q_{\infty }}=1-e^{-{\left(\frac{t-t_{0}}{\tau_{d}}\right)}^{\beta }}$$

(5) $$f_{1}=\frac{3}{2}\left[1-{\left(1-\frac{M_{t}}{M_{\infty }}\right)}^{\frac{2}{3}}\right]-\frac{M_{t}}{M_{\infty }}=kt$$

where *Q_t_/Q_∞_* is the fraction of the active compound released until time *t*, *K_K_* is the Korsmeyer constant, *n* is the release exponent (a parameter that defines the release mechanism), *β* is the shape parameter of the curve, and *k* corresponds to the slope [46,51].

### 3.5. Microencapsulation Efficiency

The encapsulation efficiencies (EE) of EGCG and/or Vit B12 into the microstructures were determined considering the release and a mass balance. This efficiency is defined as the ratio of the total amount of catechin and/or vitamin contained within the microstructures to the total amount of active ingredient used in the electrospinning process (Equation (6)).

To determine the amount of EGCG/vitB12 encapsulated within the microstrutures, two assumptions were made: the amount of EGCG/VitB12 that was not encapsulated was assumed to be the amount released immediately (at time zero) during release assays, and the total amount of EGCG/VitB12 released at the end of the release assays was considered to include the total amount of EGCG/VitB12 (inside and outside the microstructures). The encapsulation efficiency can be calculated from the graphs that represents the amount of active substance released during the release assays.

(6) $$\mathrm{EE}\;\left(\%\right)=\frac{\mathrm{Total}\;\mathrm{amount}\;\mathrm{released}-\mathrm{Amount}\;\mathrm{released}\;\mathrm{in}\;\mathrm{time}\;\mathrm{zero}}{\mathrm{Total}\;\mathrm{amount}\;\mathrm{released}}\times 100$$

### 3.6. Statistical Analysis

All the analytical determinations were made in triplicate, and the results were expressed with standard deviations associated to the measures. The results of statistical significance were analyzed (at a level of significance *p* ≤ 0.05) by a single-factor analysis of variance (ANOVA) and Tukey’s test.

## 4. Conclusions

The purpose of this study was to produce microstructures loaded with EGCG, vitamin B12, and the mixture of both and characterize according to the morphology, release profiles, and encapsulation efficiency, which were successfully achieved.

Different structures were obtained: microparticles in the lowest concentrations of zein (1% *w*/*v* and 5% *w*/*v*) and fibers in the highest zein concentrations (30% *w*/*v*). This indicates that the concentration of encapsulating agent has a great impact in the microstructure’s morphology since this can alter the solution conductivity or increase the interaction between the biopolymer and the solvent. On the other hand, the type/concentration of bioactive compound, and the fact that the sample loading been done by pressure or pump did not affect the morphology of the final structures.

In all of the samples, it was possible to distinguish two different zones: release and stabilization zones. The slower releases were observed in the fibers, which can suggest that increasing the polymer concentration is associated with an increasing of the release time. As opposed, the samples with highest bioactive compound concentration are the ones with fastest release.

Finally, five kinetic models were adjusted to the release profiles of each sample. In general, the best model to fit the majority of results was the Weibull model. In addition, by the analysis of the release exponent, *n*, obtained from the fitting of the Korsmeyer–Peppas model, was possible to determine that samples followed either a Fickian Diffusion,” a “Super Case-II transport”(the release is entirely controlled by stress-induced relaxations) or an “Anomalous transport”(the release is controlled by Fickian diffusion and polymer chain relaxation).

Concluding, it is safe to say that vitamin B12 and EGCG were successfully encapsulated in the proposed matrix, zein, and by the technique used, electrospinning. Based on these results, it would be intriguing to further explore the potential applications of these microstructures the industry. To do so, it may be useful to replicate the materials and methods used in this study and employ additional analysis techniques to evaluate their feasibility for such as food, cosmetics, and biomedical fields.

## Figures and Tables

**Table 1:** Parameters and correlation coefficients of each equation from the kinetic models of the zein microstructures loaded with EGCG or VitB12. Samples marked with * was not possible to fit any model due to the fast core release.

SAMPLE	Zero Order		First Order		Baker-Lonsdale		Korsmeyer-Peppas			Weibull		
	*K_o_*	R^2^	*K*_1_	R^2^	*K*	R^2^	*K_k_*	*n*	R^2^	*τ_d_* (min)	*β*	R^2^
Z:1:E:1	*											
Z:5:E:1	*											
Z:15:E:1	5.69 × 10^−2^	0.765	−6.99 × 10^−2^	0.738	9.74 × 10^−2^	0.845	0.738	3.16 × 10^−2^	0.634	0.6	0.403	0.761
Z:30:E:1	9.91 × 10^−2^	0.779	−0.804	0.442	1.86 × 10^−2^	0.970	0.147	0.956	0.789	5.1	0.519	0.897
Z:30:E:5	7.13 × 10^−2^	0.861	−0.143	0.776	2.35 × 10^−2^	0.966	0.570	0.124	0.917	3.0	0.439	0.959
Z:30:E:0.5	9.41 × 10^−2^	0.824	−0.381	0.660	1.34 × 10^−2^	0.996	0.342	0.310	0.990	6.7	0.518	0.972
Z:1:B:1	1.60 × 10^−3^	0.889	−0.198	0.750	3.27 × 10^−2^	0.926	0.446	0.203	0.729	3.4	0.991	0.918
Z:5:B:1	*											
Z:15:B:1	0.144	0.780	−0.249	0.664	6.26 × 10^−2^	0.992	0.756	0.135	0.877	1.4	0.681	0.765
Z:30:B:1	1.80 × 10^−3^	0.970	−1.167	0.462	1.61 × 10^−2^	0.981	6.76 × 10^−2^	1.115	0.919	6.5	1.051	0.994
Z:30:B:5	0.109	0.985	−0.999	0.587	1.34 × 10^−2^	0.993	8.83 × 10^−2^	0.970	0.905	8.0	0.970	0.979
Z:30:B:0.5	9.37 × 10^−2^	0.956	−0.600	0.536	1.72 × 10^−2^	0.978	8.66 × 10^−2^	1.046	0.933	6.9	0.959	0.959

**Table 2:** Parameters and correlation coefficients of each equation from the kinetic models of the zein microstructures loaded with (EGCG + VitB12). Samples marked with * was not possible to fit any model due to the fast core release.

SAMPLE	Zero Order				First Order				Baker–Lonsdale			
	*Ko*		R^2^		*K*_1_		R^2^		*K*		R^2^	
	EGCG	B12	EGCG	B12	EGCG	B12	EGCG	B12	EGCG	B12	EGCG	B12
Z:1:EB:1	0.056	0.0726	0.887	0.850	−0.168	0.159	0.765	0.725	0.008	0.026	0.976	0.969
Z:5:EB:1	*											
Z:15:EB:1	0.109	0.096	0.849	0.847	−0.872	0.350	0.557	0.705	0.006	0.014	0.978	0.991
Z:30:EB:1	0.037	0.0413	0.974	0.975	−0.139	0.144	0.867	0.893	0.005	0.007	0.992	0.978
Z:30:EB:5	0.147	0.1863	0.969	0.976	−0.807	0.763	0.775	0.516	0.011	0.03	0.989	0.998
Z:30:EB:0.5	0.105	0.0465	0.930	0.933	−0.313	0.285	0.559	0.516	0.007	0.007	0.997	0.987
**SAMPLE**	**Korsmeyer** **–** **Peppas**						**Weibull**					
	***K_k_***		***n***		**R^2^**		***τ_d_*** **(min)**		***β***		**R^2^**	
	**EGCG**	**B12**	**EGCG**	**B12**	**EGCG**	**B12**	**EGCG**	**B12**	**EGCG**	**B12**	**EGCG**	**B12**
Z:1:EB:1	0.316	0.428	0.282	0.238	0.943	0.83	10.7	3.5	0.545	0.526	0.957	0.883
Z:5:EB:1	*											
Z:15:EB:1	0.169	0.186	0.495	0.605	0.912	0.88	13.6	6.5	0.528	0.488	0.977	0.958
Z:30:EB:1	0.063	0.058	0.766	0.863	0.929	0.950	21.8	16.6	0.894	0.929	0.977	0.979
Z:30:EB:5	0.291	0.375	0.364	0.339	0.973	0.964	7.7	3.2	0.509	0.610	0.987	0.971
Z:30:EB:0.5	0.048	0.038	0.961	1.128	0.860	0.898	15.5	11.2	0.707	0.784	0.978	0.977

**Table 3:** Entrapment efficiency for the microstructures produced by electrospinning.

SAMPLE	EE (%)	SAMPLE	EE (%)	SAMPLE		EE (%)
Z:1:E:1	19.26 ± 0.08	Z:1:B:1	108.7 ± 0.4 *	Z:1:EB:1	EGCG	92.4 ± 0.4
					B12	70.5 ± 0.3
Z:5:E:1	0.309 ± 0.001	Z:5:B:1	89.7 ± 0.4	Z:5:EB:1	EGCG	47.6 ± 0.3
					B12	86.6 ± 0.4
Z:15:E:1	39.75 ± 0.16	Z:15:B:1	81.1 ± 0.3	Z:15:EB:1	EGCG	99.7 ± 0.4
					B12	78.6 ± 0.3
Z:30:E:1	100.0 ± 0.4	Z:30:B:1	93.9 ± 0.4	Z:30:EB:1	EGCG	100.0 ± 0.4
					B12	100.0 ± 0.4
Z:30:E:5	74.3 ± 0.3	Z:30:B:5	79.6 ± 0.3	Z:30:EB:5	EGCG	97.2 ± 0.4
					B12	105.1 ± 0.4 *
Z:30:E:0.5	95.6 ± 0.4	Z:30:B:0.5	77.1 ± 0.3	Z:30:EB:0.5	EGCG	103.2 ± 0.4 *
					B12	96.6 ± 0.4

* above 100% due to analytical errors and interferences in the absorbance measurement.

**Table 4:** Description of the solution conditions tested in electrospinning process for the samples containing EGCG and the samples containing Vitamin B12.

Biopolymer	Active Ingredient	Biopolymer Concentration (% *w*/*v*)	Active Ingredient Concentration (% *w*/*w*)	SAMPLE	Flow Rate (mL/h)
Zein	EGCG	1	1	Z:1:E:1	0.6
		5	1	Z:5:E:1	0.3
		15	1	Z:15:E:1	0.6
		30	1	Z:30:E:1	3
		30	5	Z:30:E:5	1.5
		30	0.5	Z:30:E:0.5	1.5
	Vitamin B12	1	1	Z:1:B:1	0.6
		5	1	Z:5:B:1	0.3
		15	1	Z:15:B:1	0.6
		30	1	Z:30:B:1	3
		30	5	Z:30:B:5	1.5
		30	0.5	Z:30:B:0.5	1.5
	EGCG + Vitamin B12	1	0.5 + 0.5	Z:1:EB:1	0.6
		5	0.5 + 0.5	Z:5:EB:1	0.3
		15	0.5 + 0.5	Z:15:EB:1	0.6
		30	0.5 + 0.5	Z:30:EB:1	3
		30	2.5 + 2.5	Z:30:EB:5	1.5
		30	0.25 + 0.25	Z:30:EB:0.5	1.5

## Data Availability

Data that support the findings of this study are available on request to the authors.
